# Pemphigoid diseases as immune-related adverse effect following immune checkpoint inhibitors: A clinical case series of a diverse spectrum

**DOI:** 10.1016/j.jdcr.2025.03.021

**Published:** 2025-04-04

**Authors:** Nika Kotnik, Gilles F.H. Diercks, Hilde Jalving, Geke A.P. Hospers, Sjoukje F. Oosting, Michel van Kruchten, T. Jeroen N. Hiltermann, Maria C. Bolling, Jeroen Bremer, Barbara Horváth, Joost M. Meijer

**Affiliations:** a Department of Dermatology, University of Groningen, University Medical Center Groningen, UMCG Center of Expertise for Blistering Diseases, European Reference Network for Rare Skin Diseases (ERN SKIN), Groningen, the Netherlands; b Division of Experimental Allergy and Immunodermatology, University of Oldenburg, Oldenburg, Germany; c Department of Pathology, University of Groningen, University Medical Center Groningen, Groningen, the Netherlands; d Department of Medical Oncology, University of Groningen, University Medical Center Groningen, Groningen, the Netherlands; e Department of Pulmonary Diseases and Tuberculosis, University of Groningen, University Medical Center Groningen, Groningen, the Netherlands

**Keywords:** autoimmune bullous disease, bullous pemphigoid, checkpoint inhibitor, immune-related adverse effect, immunotherapy

## Introduction

Cutaneous immune-related adverse effects (irAEs) are the most frequently reported irAEs and occur in up to 40% to 60% of patients with a malignancy treated with immune checkpoint inhibitors (ICIs).^1^ With the immunomodulatory effect of ICIs, the generated immune response may lead to adverse autoimmunity, such as bullous pemphigoid (BP).^2^, ^3^, ^4^, ^5^, ^6^ However, other pemphigoid subtypes may present as pruritus without skin blistering (nonbullous pemphigoid, NBP)^7^ or affecting predominantly mucosa (mucous membrane pemphigoid, MMP).^8^^,^^9^ In this case series, we describe 12 patients with a broad clinical spectrum of pemphigoid diseases associated with ICI treatment and report clinical features, diagnostic findings, and impact on treatment.

## Methods

In this retrospective monocenter case series, patients were included with confirmed diagnosis of pemphigoid disease with during or after treatment with PD-1 inhibitors from February 2019 to December 2023.^10^ Patient data were collected on demographic, clinical, histopathological, immunofluorescence, and serological data. No ethical review committee approval is needed for this type of research according to national guidelines, informed consent was obtained.

## Case series

In total 12 patients (median age 69 years [range 60-80]; 75% male) were identified who developed various subtypes of pemphigoid diseases as irAE during or after treatment with PD-1 inhibitors nivolumab and pembrolizumab, with a median time to onset of pemphigoid disease of 8.8 months [range 1.5-24] (Table I). Clinical subtypes included BP (*n* = 3), NBP (*n* = 4), lichen planus pemphigoides (*n* = 2), MMP (*n* = 1), and Brunsting-Perry cicatricial pemphigoid (*n* = 1). All pemphigoid irAE were considered as severe irAE (grade 3-4) according to Common Terminology Criteria for Adverse Events (v5.0).^11^ ICI treatment was discontinued in 8 of 12 patients (75%); in 7 cases due to cutaneous irAE, in 1 case due to concomitant pneumonitis. The remaining 4 patients had completed ICI treatment cycle of 12 or 24 months before onset of pemphigoid, with partial or complete response (Table I). During follow-up (mean 25.5 months) 2 patients died, due to progression of malignancy or complications. Here, we illustrate 4 cases of various pemphigoid diseases as irAE and report characteristics and cases number 5-12 in Table I.

**Table I:** Patient characteristics, diagnostics, treatment, and outcome of immune-checkpoint inhibitor associated pemphigoid diseases

Patient	Diagnosis	ICI	Malignancy	ICI treatment	Time to onset pemphigoid	Clinical features	Histopathology (skin biopsy)	DIF BMZ (skin biopsy)	IIF SSS	ELISA (u/mL) and immunoblot (IB)	Treatment response pemphigoid	Follow-up after diagnosis pemphigoid	Outcome malignancy
1/M/62	BP	P	Urothelial cell carcinoma	Discontinued due to irAE	4 wk	Pruritus, urticarial plaques and tense blisters upper body	Subepidermal split with eosinophilic infiltrate	Linear n-serrated IgG 2+, C3c 3+	IgG 1+ roof	BP180 NC16A 143, IB BP180 pos	CR OCS 0.75 mg/kg/d + HIVIG 2g + RTX 2 × 1000 mg iv	3 mo	Death due to disease progression
2/M/80	NBP	N	Squamous cell carcinoma lung	2 y cycle completed	4 mo	Generalized pruritus, erythematous papules, excoriations	Interface dermatitis	Linear Ig+, C3c +	IgG 2+ roof	BP180 NC16A 31, BP230 38, IB BP180 pos	CR lesional clobetasol cream	46 mo	Alive, sustained complete response
3/F/65	MMP	N	Malignant melanoma	Discontinued due to irAE	11 mo	Vesicles and blisters oral mucosa, pruritic erythematous papules on skin	Interface dermatitis with eosinophilic infiltrate	Mucosa: linear n-serrated IgG 2+, C3c 3+; Skin: linear n-serrated IgG2+	IgG 2+ roof	Neg	CR dapsone 50-100 mg/d + clobetasol cream	34 mo	Alive, sustained complete response
4/M/74	B-PP	N	Renal cell carcinoma	Discontinued due to irAE	18 mo	Delayed wound healing, pruritus, vesicles and blisters on skin graft on the scalp, neck and donor site	Subepidermal split with eosinophilic infiltrate	Linear n-serrated IgG3+, IgA +/−	IgG3+ roof	BP180 NC16A 16, IB BP180 pos	CR OCS 0.75 mg/kg/d + whole body clobetasol cream, doxycycline, RTX 2 × 1000 mg iv	36 mo	Alive, sustained partial response
5/M/71	BP	P	Adenocarcinoma lung	2 y cycle completed	24 mo	Pruritus, extensive erythema, blisters and vesicles	Subepidermal split with eosinophilic infiltrate	Linear IgG+, C3c+	IgG 3+ roof	BP180 NC16A 100, IB BP180 pos	CR whole body clobetasol cream	45 mo	Alive, recurrence after 39 mo, restart pembrolizumab without recurrence of BP, progression after 8 cycles, palliative chemotherapy
6/M/73	NBP	P	Adenocarcinoma lung	Discontinued due to irAE	5 mo	Generalized pruritus, erythematous papules, eczematous plaques and excoriations	Interface dermatitis with a perivascular lymphocytic infiltrate	Linear IgG3+ (2x)	Neg	Neg	CR OCS 2 mg/kg/d + whole body clobetasol cream	9 mo	Death due to pulmonary hemorrhage, obduction no tumor left, ypT0N0M0
7/F/64	NBP	N	Malignant melanoma	Discontinued due to complete response and pneumonia	14 mo	Pruritic erythematous papules on trunk and extremities	Eosinophilic dermatitis with spongiosis	Linear n-serrated IgG 2+, C3c 1+ (2x)	Neg	Neg	CR whole body clobetasol cream	34 mo	Alive, sustained complete response
8/M/63	LPP	P	Urothelial cell carcinoma	Combined enfortumab vedotin, discontinued due to irAE	11 mo	Pruritic violaceus papules with Wickham striae/tense blisters, and blisters on normal appearing skin	Interface dermatitis, subepidermal split with eosinophilic spongiosis	Negative BMZ, positive colloid bodies∗	IgG 2+ roof	Neg, IB BP180 pos	PR OCS 0.8 mg/kg/d + doxycycline 100 mg/d + lesional clobetasol cream	15 mo	Alive, sustained partial response
9/M/77	NBP	P	Squamous cell carcinoma lung	Combined carboplatin/paclitaxel, 2 year cycle completed	5 mo	Disseminated pruritic erythematous papules and plaques	Eosinophilic dermatitis with spongiosis	Linear n-serrated IgG 3+, C3c 1+	IgG 1+ roof	Neg, IB BP180 pos	CR whole body clobetasol cream	23 mo	Alive, sustained partial response
10/F/78	LPP	N	Renal cell carcinoma	Discontinued due to irAE	5 mo	Disseminated pruritic polygonal papules with Wickham striae on wrists, arms, legs and feet and vesicles. Buccal mucosa Wickham striae.	Interface dermatitis with a lymphocytic infiltrate and Civatte bodies	Linear n-serrated and granular IgG 2+, C3c 2+	IgG 2+ roof	Neg, IB BP180 pos	CR OCS 0.4 mg/kg/d + clobetasol cream	18 mo	Alive, sustained partial response
11/M/61	BP	N	B-cel lymphoma	1 year cycle completed, complete response	4 mo	Disseminated pruritic urticarial plaques with tense blisters	Subepidermal split with eosinophilic infiltrate	Linear n-serrated IgG 2+, C3c 1+	IgG 1+ roof	BP180 NC16A 99, IB BP180 pos	PR OCS 0.5 mg/kg/d + clobetasol cream, MTX 10-20 mg/wk	29 mo	Alive, sustained complete response
12/M/60	LPP	N	Malignant melanoma	Discontinued due to cirAE and increased troponin	3 mo	Polygonal papules and plaques with Wickham striae, tense blisters on normal appearing skin	Interface dermatitis, subepidermal split with eosinophilic spongiosis	Linear n-serrated IgG 2+	IgG 1+ roof	Neg, IB BP180 pos	CR daily lesional clobetasol cream	8 mo	Alive, sustained complete response

*BMZ*, Basement membrane zone; *B-PP*, Brunsting-Perry pemphigoid; *BP*, bullous pemphigoid; *C3c*, complement C3; *CR*, complete remission; *DIF*, direct immunofluorescence microscopy; *ELISA*, enzyme-linked immuno sorbent assay; *HIVIG*, human intravenous immunoglobulin; *ICI*, immune-checkpoint inhibitor; *IgG*, immunoglobulin G; *IIF SSS*, indirect immunofluorescence salt-split skin; *irAE*, immune-related adverse effect; *LLP*, lichen planus pemphigoide; *MMP*, mucous membrane pemphigoid; *MTX*, methotrexate; *N*, nivolumab; *NBP*, nonbullous pemphigoid; *P*, pembrolizumab; *PR*, partial remission; *RTX*, rituximab anti-CD20 mAbN.

∗ Immunofluorescence during high-dose prednisolone.

### Case 1 BP

**Fig 1:**
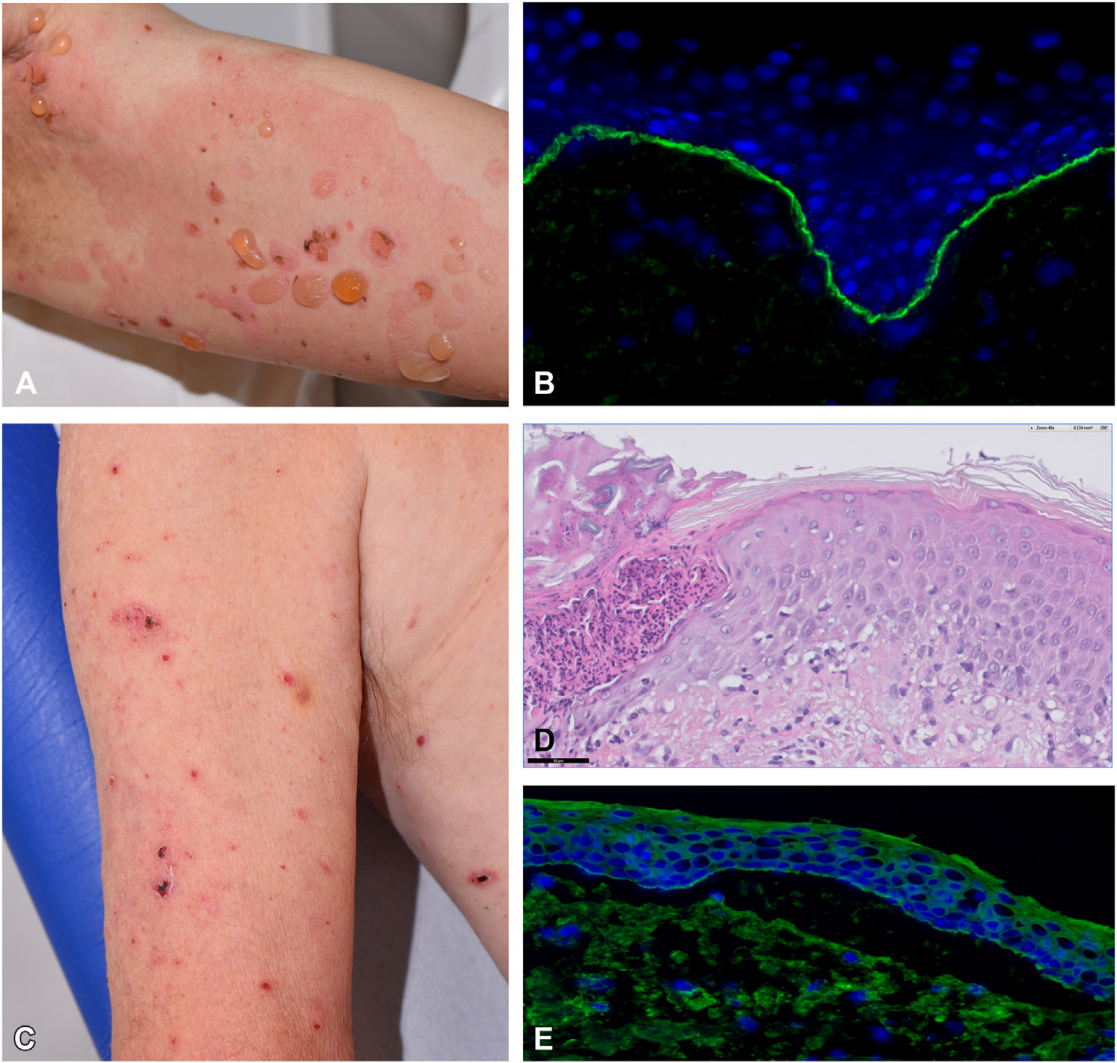
Clinical, histopathological, and immunofluorescence findings of bullous and nonbullous pemphigoid associated with immune-checkpoint inhibitors (ICIs). **A,** Tense bullae on erythematous skin in bullous pemphigoid (BP) (patient number 1). **B,** Intense linear deposition of IgG along the basement membrane zone (BMZ) (patient number 5). **C** and **D,** Excoriated erythematous papules in nonbullous pemphigoid (patient number 2) in with histopathology of a lichenoid dermatitis (H-E staining). **E,** Positive serology in patient number 2 with epidermal side staining of IgG using indirect immunofluorescence (IIF) on salt-split skin substrate.

A 62-year-old patient experienced an acute onset of pruritus, urticarial papules and extensive tense blisters on the arms, chest, and back (Fig 1, *A*) after the second cycle of pembrolizumab for disseminated urothelial carcinoma. Mucosa was not affected. A diagnosis of BP was confirmed by direct immunofluorescence (DIF) (Fig 1, *B*) and indirect immunofluorescence on salt-split skin (IIF, Table I). Pembrolizumab was discontinued and treatment initiated with oral corticosteroids (prednisone up to 0.75 mg/kg/day), whole-body application of superpotent topical corticosteroids (30 gram/day), and the addition of Rituximab 2× 1000 mg intravenous and human intravenous immunoglobulins (2 gram/kg divided over 3 days) led to complete remission of BP. The patient died 4 months after diagnosis of BP, due to progression of disseminated urothelial carcinoma.

### Case 2 NBP

A 80-year-old patient with a history of disseminated squamous cell carcinoma of the lung received 52 cycles of treatment with nivolumab, following chemotherapy. Four months after initiation of nivolumab the patient developed mild pruritic papules. After nearly 2 years of nivolumab treatment, the skin complaints increased to generalized pruritus and widespread pruritic erythematous papules and excoriations (Fig 1, *C*). No skin blistering or mucosal lesions were observed. Histopathologic analysis of the pruritic papules showed an interface dermatitis without eosinophils (Fig 1, *D*). However, DIF findings revealed positive IgG (1+) and complement C3 (1+) along the epidermal basement membrane zone, combined with epidermal side staining of IgG (2+) by IIF salt-split skin (Fig 1, *E*) and positive serology (Table I). A diagnosis of NBP was confirmed and complete remission achieved with whole-body application of superpotent topical corticosteroids. Nivolumab remained discontinued, because the patient completed 2 years of treatment.

### Case 3 MMP

**Fig 2:**
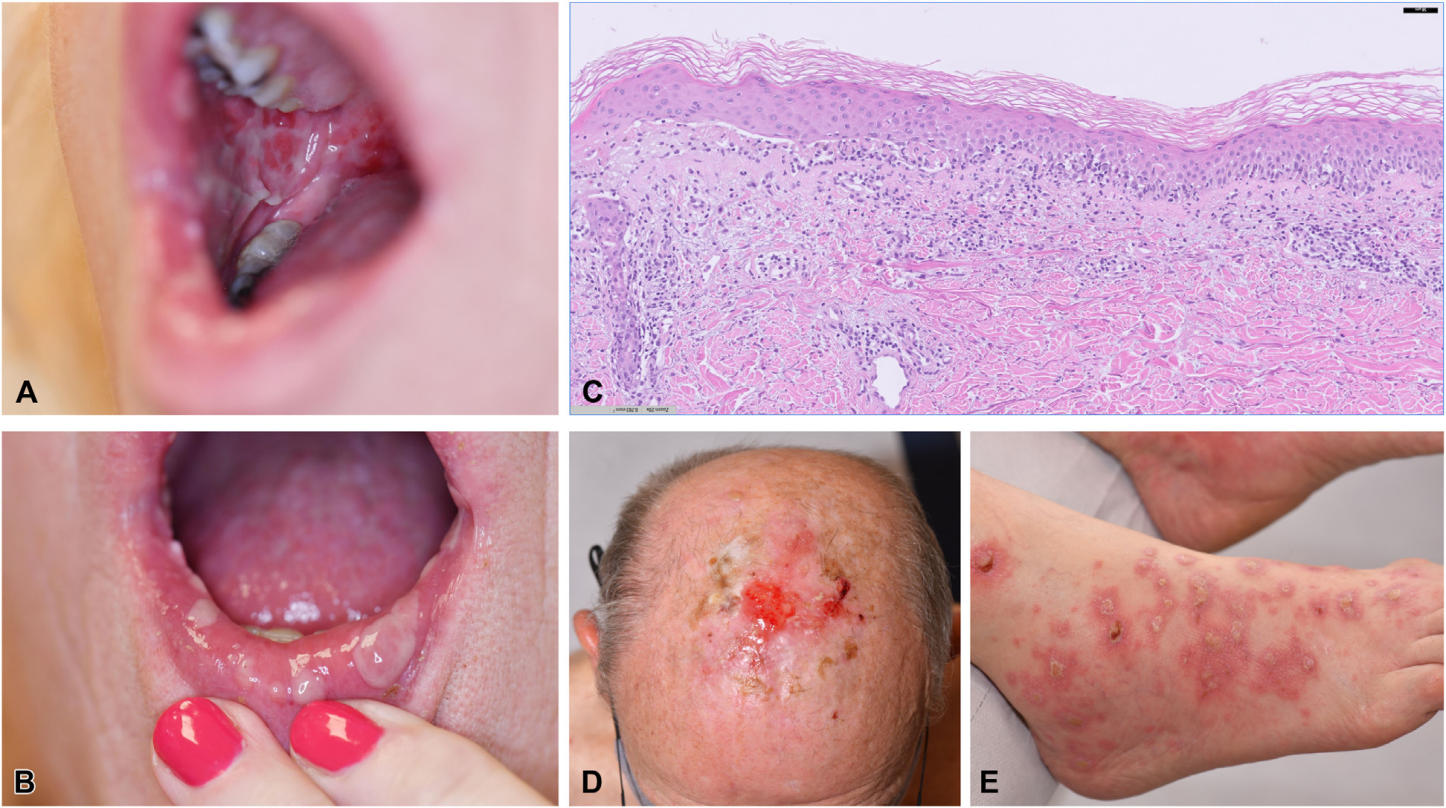
Clinical, histopathological, and immunofluorescence findings of mucous membrane pemphigoid, Brunsting-Perry pemphigoid, and lichen planus pemphigoides associated with immune-checkpoint inhibitors (ICIs). **A** and **B,** Oral blisters and erosions on the buccal mucosa and lips in patient number 3 with mucous membrane pemphigoid (MMP). **C,** Histopathology (H-E staining) of a skin biopsy revealing an interface dermatitis with eosinophils and a subtle subepidermal split (patient number 3). **D,** Localized erosions and blisters on the scalp and delayed wound healing with scarring after skin grafting in Brunsting-Perry pemphigoid (patient number 4). **E,** Lichen planus and blisters on the foot in lichen planus pemphigoides (patient number 10).

A 65-year old patient with disseminated melanoma was treated with nivolumab and endured pruritus after each cycle of treatment. After 14 cycles of treatment the patient developed vesicles and blisters on the oral mucosa and pruritic erythematous papules on the skin (Fig 2, *A* and *B*). A herpes simplex polymerase chain reaction was negative. Histopathology of a mucosal biopsy showed ulcerative inflammation, a skin biopsy revealed an interface dermatitis with eosinophils and a subtle subepidermal split (Fig 2, *C*). DIF and serology confirmed a diagnosis of MMP (Table I). Because of the acute onset the patient was treated with oral and topical corticosteroids leading to partial remission, successful adjunctive treatment with dapsone was initiated. However, treatment with nivolumab remained discontinued.

### Case 4 Brunsting-Perry pemphigoid

A 74-year-old patient was diagnosed with disseminated renal carcinoma 7 years ago and has been receiving treatment with nivolumab for nearly 1.5 years, with a stable partial response. The patient reported pruritus since the initiation of nivolumab. Upon presentation, the patient exhibited pruritus, along with vesicles, blisters, and ulcerative plaques on a skin graft on the scalp following the surgical excision of metastasis. Moreover, also the donor site on the right leg was affected (Fig 2, *D*). The patient’s history revealed delayed wound healing and significant scarring at both the donor and recipient sites after the skin graft. Additionally, mucosal involvement was noted, with hemorrhagic nasal erosions and crusting. Histopathology showed subepidermal splits and eosinophils, while DIF revealed linear IgG deposition (n-serrated) along the epidermal basement membrane zone. Serology confirmed IgG antibodies against BP180, leading to the diagnosis of Brunsting-Perry pemphigoid with localized cicatricial lesions primarily affecting the head and neck. Treatment with nivolumab was discontinued, and therapy was initiated with oral corticosteroids and whole-body application of superpotent topical corticosteroids, resulting in complete remission.

## Discussion

In this case series, we report a broad clinical spectrum of pemphigoid diseases associated with ICI treatment: with or without skin blisters, affecting skin and/or mucosa, concomitant with lichenoid features (Fig 2, *E*) or triggered by surgery. The recognition of the nonbullous variant of pemphigoid suggests pemphigoid should also be considered in refractory pruritic cutaneous eruptions during or after ICI treatment.^7^^,^^10^

Prior studies reviewed clinical features associated with ICI-induced BP, with prodromal symptoms before onset of BP in half of the patients, pruritus the most common symptom (97.7%), and onset of BP even after discontinuation of ICI treatment.^12^ In addition, we report the frequent histopathological findings of an interface dermatitis in NBP, MMP, and lichen planus pemphigoides, which can be misdiagnosed as lichenoid drug-reaction. Moreover, less frequent circulating IgG to the NC16A domain of BP180 was detected by enzyme-linked immunosorbent assay, while confirmation of anti-BP180 IgG by immunoblot suggests different epitopes of BP180 may be targeted.

In a recent review by Asdourian et al., Common Terminology Criteria for Adverse Event grading of ICI-induced BP was classified as grade 3 in 35.1% (*n* = 39) and grade 4 in 15.3% (*n* = 17) of patients, leading to ICI discontinuation in 58.3%.^12^ Interestingly, ICI treatment was discontinued and reintroduced in 7 reported patients, and successfully continued in 20 patients.^12^ In our case series, in 1 patient ICI treatment was reintroduced after remission of BP without recurrence of BP. A cohort analysis suggested that development of cutaneous irAEs is associated with response to ICI treatment and patient survival (BP, hazard ratio 0.524), which warrants future research.^13^

## Conclusions

Pemphigoid diseases as irAE during ICI treatment may present with heterogeneous clinical features; with or without skin blisters, affecting skin or mucosa and concomitant with lichen planus. ICI-induced pemphigoid diseases significantly impact quality of life and may impact on discontinuation of ICI treatment. In this respect, we encourage to perform diagnostic tests for pemphigoid at an early stage in patients with suspected mucocutaneous irAEs.

## Conflicts of interest

None disclosed.
